# Current Challenges in Diagnosis of Venous Thromboembolism

**DOI:** 10.3390/jcm9113509

**Published:** 2020-10-29

**Authors:** Zachary Liederman, Noel Chan, Vinai Bhagirath

**Affiliations:** 1 Department of Medicine, University of Toronto, Toronto, ON M5S 1A8, Canada; 2 Department of Medicine, University Health Network, Toronto, ON M5G 2C4, Canada; 3 Population Health Research Institute, Hamilton, ON L8S 4L8, Canada; chanwankai@HHSC.CA; 4 Thrombosis and Atherosclerosis Research Institute, Hamilton, ON L8L 2X2, Canada; bhagiv@mcmaster.ca; 5 Department of Medicine, McMaster University, Hamilton, ON L8S 4L8, Canada

**Keywords:** venous thromboembolism, diagnosis, deep-vein thrombosis, pulmonary embolism

## Abstract

In patients with suspected venous thromboembolism, the goal is to accurately and rapidly identify those with and without thrombosis. Failure to diagnose venous thromboembolism (VTE) can lead to fatal pulmonary embolism (PE), and unnecessary anticoagulation can cause avoidable bleeding. The adoption of a structured approach to VTE diagnosis, that includes clinical prediction rules, D-dimer testing and non-invasive imaging modalities, has enabled rapid, cost-effective and accurate VTE diagnosis, but problems still persist. First, with increased reliance on imaging and widespread use of sensitive multidetector computed tomography (CT) scanners, there is a potential for overdiagnosis of VTE. Second, the optimal strategy for diagnosing recurrent leg deep venous thrombosis remains unclear as is that for venous thrombosis at unusual sites. Third, the conventional diagnostic approach is inefficient in that it is unable to exclude VTE in high-risk patients. In this review, we outline pragmatic approaches for the clinician faced with difficult VTE diagnostic cases. In addition to discussing the principles of the current diagnostic framework, we explore the diagnostic approach to recurrent VTE, isolated distal deep-vein thrombosis (DVT), pregnancy associated VTE, subsegmental PE, and VTE diagnosis in complex medical patients (including those with impaired renal function).

## 1. Introduction

Venous thromboembolism (VTE) consists of deep-vein thrombosis (DVT) and/or pulmonary embolism (PE), and occurs in approximately 1 in 1000 persons per year [1,2]. Prompt diagnosis and initiation of therapy is vital, since untreated PE has a mortality rate of approximately 30%, and nearly 30% of untreated DVTs will result in severe swelling or ulceration of the leg [1,2]. With prompt diagnosis and treatment, PE- or treatment-related death is less than 1% [3]. Further, accurately ruling out VTE avoids unnecessary treatment with anticoagulation and the attendant risks.

The paradigm of VTE diagnosis is to accurately identify all patients who would benefit from treatment as quickly as possible while minimizing the burden of diagnostic tests performed on patients without VTE. In recent decades, advances in diagnostic strategies have helped to reduce the number of imaging tests required in patients with suspected VTE, while missing few patients who would have benefited from treatment. These diagnostic strategies for VTE can be considered in several phases—first the patient’s history and physical examination lead the clinician to suspect VTE; second, screening tests to rule out VTE may be performed; and finally, patients in whom VTE cannot be ruled out undergo a more definitive imaging study. In this review, we describe the current approaches to diagnosis of VTE and focus on areas that require improvement.

### 1.1. Initial Suspicion of VTE

The initial suspicion of PE or DVT relies on clinical *gestalt*, with no guideline-based recommendations on when to consider VTE in a undifferentiated population. Missed diagnosis of PE remains one of the most common causes of medical harm and litigation [4], indicating a need for better understanding of when to suspect this diagnosis. Diagnostic pathways for hospital inpatients are especially poorly defined.

Once VTE is suspected, several studies have investigated the predictive value of various signs, symptoms, comorbidities, and combinations thereof. No individual symptom or sign is sufficiently predictive to either rule in or rule out VTE [5,6]. However, the *gestalt* impression remains an important component of a widely used clinical prediction rule (“PE is the most likely diagnosis” in the Wells PE score, see below).

### 1.2. Clinical Prediction Rules

Clinical prediction rules, devised by combining several clinical features together, offer improved diagnostic accuracy over any sign or symptom considered in isolation (Table 1). The best validated and most widely used are the Wells PE score, the Geneva PE score, and the Wells DVT score [7]. When applied to an emergency department population with a high incidence for VTE (approximately 20%), patients identified by clinical prediction rules as being at high risk of VTE had the diagnosis confirmed by imaging 40% of the time for PE, and 85% for DVT. Conversely, the absence of suggestive clinical features can identify patients with <5% prevalence of either DVT or PE [8,9]. Despite the improved sensitivity and specificity, for most cases clinical prediction tools are unable to rule in or rule out VTE on their own. The value of clinical prediction rules is therefore in determining the most efficient next step in a diagnostic algorithm by stratifying cases into high and low clinical probabilities. For example, for low-probability patients, clinical prediction rules reduce the number of required imaging studies.

### 1.3. Biomarkers

Biomarkers can bolster clinical assessment and further reduce the need for diagnostic imaging studies. Seminal studies have established the role of D-dimer in ruling out VTE in patients with low pretest clinical probability, with more recent studies focusing on refining the cut offs used to rule out VTE [15,16]. Numerous other biomarkers have also been investigated with variable success. This includes P-selectin, thrombin generation, and C-reactive protein [17]. Currently, D-dimer remains the only biomarker in routine clinical use.

### 1.4. Imaging Tests

For patients in whom VTE cannot be reliably excluded using clinical prediction rules and D-dimer (approximately 70% of patients with standard D-dimer strategy) [14], diagnostic imaging studies are required. Current diagnostic imaging techniques, including CT pulmonary angiography (CTPA), V/Q scanning, and compression ultrasound, have a high sensitivity and specificity [15]. Sensitivity/Specificity is 93% (88–96%)/98% (96–99%) for CTPA and 96% (91–98%)/95% (89–98%) for VQ scan (high probability scan interpreted as positive, normal scan interpreted as negative), respectively [18]. Compression ultrasound is the most commonly used imaging modality for DVT. A recent meta-analysis reported sensitivity and specificity of 90.1% and 98.5%, 94.0% and 97.3%, and 97.9% and 99.8% for proximal leg, whole-leg, and serial compression ultrasound, respectively [19]. V/Q scanning has the potential to rule in or rule out PE, but a high proportion of scans will be non-diagnostic. SPECT V/Q techniques reduce the proportion of non-diagnostic scans, but large-scale clinical management studies have not been performed [20]. CTPA has a lower frequency of non-diagnostic scans, is often more easily available, and faster to perform compared to V/Q. The positive predictive value for patients with high pretest probability is approximately 96% [21]. V/Q scans and CTPA come with risks of radiation, with additional risk of contrast administration for CT; and all imaging techniques incur cost to the healthcare system and potential inconvenience for the patient.

Overall, there are many available diagnostic tools within the VTE framework and no universal “one size fits all” strategy. The clinician faced with diagnosing VTE must organize testing to strike the best balance between diagnostic power, resource stewardship and potential adverse effects for their patient. With this in mind, we present the following cases to explore approaches to VTE diagnosis in areas where standard approaches may not exist, or where current evidence is insufficient.

## 2. Main Body

### 2.1. Case 1

A 43-year-old male presents with acute on chronic swelling of his left ankle and distal calf. He has a complex past medical history significant for Crohn’s disease, hypertension, obesity and provoked isolated distal DVT. The isolated distal DVT occurred 8 years earlier and was diagnosed 3 weeks following hemicolectomy for Crohn’s disease exacerbation. He received three months of anticoagulation with Rivaroxaban 20 mg po daily. He has had persistent left-leg swelling since his incident event; however, he feels that it has gotten worse over the last 2 weeks. He rates the pain as being severe today. He denies any shortness of breath or chest pain. D-dimer is measured at 1500 ng/mL.

This patient case evokes many complex questions regarding VTE diagnosis, including the use of ultrasound for diagnosis of distal DVT, the diagnosis of recurrent DVT, differentiation of acute thrombosis from post-thrombotic syndrome, and interpretation of VTE biomarkers.

#### 2.1.1. Part 1: Differentiating Chronic VTE with PTS from Acute DVT

Diagnostic evaluation should begin with consideration of the patient’s pretest probability for acute VTE. The risk of VTE in the general population is proportional to age with risk going from 1/10,000 in early adulthood to almost 1/100 in those over 80 [22]. This patient has multiple additional thrombotic risk factors (e.g., obesity and inflammatory bowel disease) that elevate his risk beyond an age adjusted baseline [23,24,25].

As in patients with suspected first VTE, no single clinical feature is sufficient to rule in or out a diagnosis of recurrent DVT. Clinical prediction rules can still be utilized to evaluate for suspected recurrent thrombosis but there is less evidence for their use than in cases of suspected first VTE. In patients with previous thrombotic events, administration of scoring systems can be complicated by the higher baseline prevalence of DVT (compared to unselected population) and presence of post-thrombotic syndrome.

Post-thrombotic syndrome occurs in up to 50% of DVT patients and, like acute DVT, can present with unilateral leg swelling, erythema and pain [26,27,28]. While PTS may wax and wane, a clinical presentation with rapid and progressive worsening of leg symptoms suggests an acute DVT. Returning to the Wells Score for DVT, in an individual patient data meta-analysis, only the modified Wells Score for DVT (extra point given for previous VTE) was deemed safe as a rule-out test for patients with prior DVT [29].

Incorporating D-dimer can further help evaluate for acute thrombosis [7]. In patients with low or moderate pretest probability, a negative D-dimer test effectively excludes VTE [30,31]; however, due to multiple other conditions that can elevate D-dimer, positive tests using standard thresholds have low specificity [32]. In cases where there is concern for recurrent DVT, a negative D-dimer still rules out a DVT but has further reduced specificity [33].

In unselected patients, a risk-adapted D-dimer approach using either age (discussed in case 3) or pretest probability adjusted cut offs, has been shown to reduce the number of false positives while maintaining sensitivity and negative predictive value [14]. The pretest probability approach uses a higher D-dimer threshold for patients with a lower pretest probability (typically 1000 ng/mL instead of 500 ng/mL), thereby ruling out DVT in a greater proportion of patients with low pretest probability [34,35]. While this has not been validated in evaluation of recurrent DVTs, patients with prior VTE were included in these studies and made up 8% and 10% of the respective study populations [34].

Ultimately, given the above challenges, many patients with prior DVT will require imaging to confirm or exclude a VTE diagnosis. Occlusion of a new venous segment or substantial extension of previous thrombus (e.g., >10 cm) should be considered as a new DVT [7,15]. Conversely, thrombus that is radiographically unchanged from prior imaging suggests chronic thrombus and post-thrombotic syndrome rather than an acute DVT. A change in residual compressed venous diameter of greater than 4 mm is generally accepted to indicate an acute DVT. A diagnostic algorithm that used a 4 mm cut off with repeat 1 week imaging (V/Q scan or lower-extremity ultrasound for borderline cases of 1–3.9 mm) was shown to be safe in a prospective cohort [36]. With this in mind, an ultrasound at the end of treatment of VTE to establish a new venous baseline should be strongly considered, as this will allow for more accurate delineation for chronic vs. acute thrombus [37]. However, even with the benefit of prior ultrasound reports, ultrasound may not be able to definitely distinguish acute from chronic thrombus; therefore, imaging should be reserved for patients with increased pretest probability for acute DVT to avoid the possibility of false positive findings.

Back to the case: A positive pretest probability adjusted D-dimer, multiple thrombotic risk factors and accelerated tempo of lower extremity symptoms, is concerning for acute VTE and necessitates an ultrasound of the affected leg. Proximal U/S of his left leg is negative with no visualized thrombus.

#### 2.1.2. Part 2: Diagnosis of Clinically Relevant Isolated Distal DVT (Below Knee)

The negative proximal U/S rules out a clinically important proximal DVT but does not exclude an isolated distal DVT (IDDVT). IDDVTs are defined as thrombus involving any lower extremity vein distal to the popliteal vein at the knee. Since up to 10% of patients with IDDVT will progress to proximal DVT or PE [38,39,40], repeat proximal U/S may be required with an initially negative proximal study. While it is tempting to proceed with imaging of the distal veins to reduce the need for serial U/S, it is important to acknowledge the limitations of whole-leg ultrasonography.

Imaging of the distal veins is more technically challenging than ultrasonography of the proximal veins and there is higher risk of false positive scans [7,41]. Compression of the proximal veins takes only a few minutes, whereas whole-leg ultrasonography is more complex and requires additional time [42]. Moreover, many institutions do not routinely offer whole-leg ultrasounds. Additionally, even a true positive IDDVT on whole-leg U/S has unclear significance as over 90% of patients with IDDVT will not progress to proximal DVT or PE [38,39,40].

If limiting ultrasound imaging to only proximal veins, anticoagulation can be safely deferred in standard-risk patients with an initial negative ultrasound pending a one-week repeat study [15,39,43]. With this approach, patients who develop interval proximal DVT on the repeat ultrasound should be treated. Conversely, patients who continue to have negative proximal ultrasounds after one week do not require anticoagulation. Most such patients do not have DVT, whereas approximately 10% have non-extending IDDVT, which rarely if ever causes PE and usually resolves spontaneously [39,43]. Due to the time requirements of whole-leg ultrasonography, serial-leg dopplers are often the more resource-efficient strategy.

In higher-risk patients (e.g., extensive thrombus or thrombus in close proximity to deep venous system, history of VTE, inpatient status, and active cancer), anticoagulation is recommended over observation for isolated distal DVT [7]. As a result, whole-leg ultrasonography may be preferred over serial proximal ultrasound. D-dimer can also be integrated into IDDVT strategies. An elevated D-dimer suggests the presence of an acute DVT (including extending IDDVT) and a normal D-dimer result identifies patients without proximal DVT and without extending IDDVT [39]. In other words, a negative D-dimer rules out DVT that has the potential for progression and embolization. In patients with a negative proximal ultrasound and normal D-dimer levels (using standard cut offs), prospective studies have shown that anticoagulation can be omitted without the need for serial ultrasound [44,45]. Completing lung imaging to identify subclinical pulmonary embolism in patients with IDDVT and positive D-dimers is an approach currently under investigation [46].

Back to the case: Since the patient is symptomatic and has an elevated D-dimer, further ultrasound is required. We favor proceeding with a whole-leg ultrasound to evaluate for a distal DVT (comparing to previous scans with first VTE) as identification of an IDDVT provides a potential therapeutic target to address the patient’s symptoms. Serial observation with repeat ultrasound in one week, remains an acceptable alternative without increased risk of VTE at 3 months.

Whole-leg ultrasound revealed partial non-compressibility of the posterior tibial vein, similar to the baseline study. The patient was reassured and given guidance on management of post-thrombotic syndrome.

### 2.2. Case 2

A 32-year-old woman in her first pregnancy presents at 20 weeks’ gestational age with shortness of breath, small-volume hemoptysis, sinus tachycardia and left-leg edema. She has no significant past medical history and the pregnancy up until the point has been uncomplicated. Chest X-ray is unremarkable.

Pregnancy increases the risk of VTE, with an overall 1–2 in 1000 rate of venous thrombosis [47,48,49]. Similar to the general population, no specific clinical sign or symptom is sufficiently specific or sensitive for diagnosing VTE. For example, shortness of breath is a common occurrence in pregnancy and can be caused by a broad differential of both physiologic and pathologic processes [50].

The LeFT score can be used to refine the pretest probability of DVT in pregnancy [12,51]. The LeFT score includes three clinical criteria: symptoms in the left leg (L); edema with a calf circumference difference ≥ 2 cm (E); and first-trimester presentation (Ft). In the derivation cohort, no DVTs were diagnosed in patients with zero of the above risk factors. Patients with one risk factor had a DVT prevalence of 8.8% and those with two or more risk factors had a DVT prevalence of 58% [12].

In patients with clinical concern for DVT, ultrasound is the recommended first imaging test, even if there is also concern for PE [52]. This takes into account the increased risk of lung imaging to both the fetus and mother during pregnancy [53]. As treatment generally does not differ between patients with DVT and PE and those with only DVT, ultrasound is a safe and accessible mechanism to inform treatment in pregnancy. In patients in whom a DVT is found, imaging for PE is generally not required in pregnancy. However, in patients being evaluated for PE without features of DVT, the diagnostic yield of screening ultrasounds is low and likely of limited utility [54].

Back to the case: The patient has clinical features of both DVT and PE. Applying the LeFT score, this patient has a score of 2 (left-leg presentation and edema), corresponding to a DVT probability of approximately 10% [51]. Based on this risk, further tests to evaluate for VTE are required. To avoid risks associated with lung imaging, bilateral proximal legs ultrasounds are performed. These are negative for DVT. A D-dimer is also performed and is 650 ng/mL.

As in the general population, pregnant patients with negative proximal venous ultrasounds appear to have a low rate of subsequent DVT in follow up [55]. However, there remains greater uncertainty in pregnancy and VTE remains a major cause of pregnancy-associated morbidity and mortality [56,57]. As a consequence, a greater burden of evidence to exclude VTE is required in pregnancy than in the general population and additional imaging after a single negative ultrasound is recommended [56]. In women where there is ongoing clinical concern for isolated DVT (i.e., no features of PE) we advocate for a repeat ultrasound at one week. MRI is an alternative approach and has been shown to identify a greater and more proximal clot burden than ultrasound in women with known DVT [58]. The clinical significance of this is unclear and does not necessitate the use of MRI over ultrasound for follow up of suspected DVT. Moreover, gadolinium does cross the placenta and is associated with risk to the fetus [59].

Back to the case: In addition to concern for DVT, there are also symptoms suggestive of PE. As lower-extremity U/S did not identify a DVT, the patient requires further evaluation to rule out PE.

Given the risks of radiation exposure with CT and V/Q strategies, the utility of D-dimer to obviate the need for chest imaging in pregnant women has been explored. D-dimer levels naturally rise during pregnancy thereby limiting the usefulness of standard D-dimer algorithms to rule out VTE in pregnancy [60]. Fortunately, the incorporation of pretest probability adjusted D-dimer levels have been shown to be a promising approach in pregnancy. The pregnancy adapted YEARS algorithm risk stratifies pregnant women with suspicion for PE by using three clinical criteria. The clinical score is then combined with a risk adjusted D-dimer to guide management. Out of 494 women with suspicion for PE and in whom DVT was excluded, VTE was ruled out in 195 women (no clinical YEARS criteria and D-dimer < 1000 ng/mL or 1–3 YEARS criteria and D-dimer < 500 ng/mL) without the need for a CT scan [61].

Despite improvements in the negative predictive ability of diagnostic algorithms, many women ultimately require lung imaging to assess for PE. Both CT angiography and V/Q scans have high sensitivity and CT angiography has high specificity for PE during pregnancy [62]. The decision between the two modalities is based primarily on availability and safety. Assuming both are available, we discuss with patients the inherent risks to both mother and fetus of each approach. We find it helpful to begin by reassuring the patient that the absolute risk of lung imaging is very low and the risk of empiric anticoagulation or missing a PE is much greater. However, the risk of imaging is not zero and the pros and cons of different modalities, including the possibility that indeterminate V/Q result may necessitate a CT angiogram, need to be considered.

Fetal radiation exposure is slightly higher with V/Q (0.5 mGy) scan than CT scan (0.1 mGy). Each mGy of fetal radiation exposure is associated with an approximately 1 in 2 million risk of fatal childhood cancer [53]. Therefore, the appreciable difference in fetal risk between CT and V/Q is exceedingly small and generally considered negligible [63,64]. The more significant issue is that there is greater maternal breast radiation with CT than V/Q scan which can potentially place the patient at increased risk for future breast cancer [53,56]. The magnitude of this risk in the context of breast shielding and other radiation reduction techniques is unclear. Based largely on this risk, the most recent guidelines from the American Society of Hematology suggest V/Q scan over CT but recognize both modalities as acceptable approaches in pregnancy [56]. However, since the prevalence of PE is higher in pregnant than non-pregnant patients, in cases where PE cannot be ruled out by V/Q scan, further imaging (e.g., CT angiography) should be obtained. This includes cases where there is a non-diagnostic V/Q scan or a mismatch between V/Q scan results and pretest probability.

Back to the case: As there are multiple clinical features for PE (clinical signs of DVT, hemoptysis and PE as most likely diagnosis) and a D-dimer above the risk-adapted threshold, the patient requires lung imaging to rule out or rule in a PE. After reviewing the pros and cons of V/Q and CTPA with the patient, a decision is made to proceed with V/Q scan. Unfortunately, V/Q scan is not available for at least 24 h. So as not to delay treatment or expose the patient to extended empiric anticoagulation, a CTPA is organized. The CT scan documents a right-sided segmental pulmonary embolism and anticoagulation is initiated with low-molecular-weight heparin.

### 2.3. Case 3

A 75-year-old female presents to hospital with a 2 day history of pleuritic chest pain and shortness of breath. Her past medical history is significant for diabetes, hypertension, chronic obstructive pulmonary disease (COPD) and hypothyroidism. Home medications include metformin, ramipiril, salbutamol and tiotropium. On initial assessment, she is found to be hypoxic and to have acute kidney injury (CrCl 20 mL/min with baseline of 50 mL/min).

Our third case expands on many diagnostic issues discussed in the prior cases and applies them to a medically complex and elderly patient. This includes the use of pretest probability, safety of lung imaging, and the potential for overdiagnosis in patients with advanced age and medical comorbidity.

Both age and renal insufficiency increase D-dimer, thereby potentially reducing the specificity of D-dimer testing. In cases with moderately elevated D-dimer levels, use of both pretest probability (PTP) adjusted D-dimer thresholds and age-adjusted D-dimer thresholds has been shown to reduce the number of imaging tests required to safely rule out PE. Further imaging can safely be omitted in patients with a low PTP according to the 7-item Wells score if the D-dimer is less than 1000 μg/L [35]. In the age-adjusted approach, the adjusted threshold is calculated as the patient’s age × 10 μg/L [65]. Therefore, for a 75-year-old patient the age adjusted D-dimer threshold would be 750 μg/L. In a prospective validation cohort of over 3000 patients, pulmonary embolism was safely excluded in an additional 331 patients using the age adjusted D-dimer threshold compared to a standard D-dimer approach [16]. In an individual patient data meta-analysis, pretest probability and age adjusted D-dimer thresholds had similar diagnostic performance [14]. A subsequent large study evaluating a pretest probability D-dimer strategy demonstrated greater utility (i.e., reduced need for diagnostic imaging) than any of the studies included in the patient data meta-analysis. These results combined with the ability to apply adapted D-dimers to both patients younger and older than 50 years of age makes a pretest probability D-dimer strategy highly appealing. However, this remains an area of ongoing study and both approaches are currently acceptable as diagnostic tools.

Back to the case: An acute pulmonary embolism is suspected and a D-dimer is performed which returns at 1200 μg/L. This is above both age and pretest probability adjusted thresholds. In light of the patient’s acute kidney injury and potential for worsening with intravenous contrast a decision is made to not proceed with CTPA. The patient is started on empiric anticoagulation with plan for a V/Q scan the next morning which is the earliest possible time.

Contrast induced acute kidney injury (CI-AKI) may occur in up to 35% of patients with reduced kidney function (eGFR < 30 mL/min/1.73 m^2^) undergoing CT scan and is a feared complication [66]. However, it is difficult to exclude confounding factors that can contribute to kidney injury and the risk of CI-AKI may be overestimated by many clinicians [67]. Furthermore, even in patients who go on to develop contrast induced kidney injury, the vast majority will have complete recovery and only a very small minority will require dialysis [68,69]. These risks must be balanced against the potential harms of withholding CT imaging. While V/Q scans do not carry a risk of kidney injury they are generally less accessible than CT scans.

Overall, in patients at risk for CI-AKI we recommend V/Q scan over a CT scan, if the V/Q scan can be obtained in a short period of time. While the diagnostic decision needs to be individualized, we feel that for uncomplicated patients with suspected VTE, it is acceptable to wait up to 12–24 h for the V/Q scan to be completed with empiric anticoagulation in the interim. This is in keeping with guideline recommendations for a timely diagnosis [18]. For patients with high bleeding risk or those who may require further therapy (e.g., submassive pulmonary embolism being assessed for tPA) we feel that the benefits of a quick diagnosis via CT generally outweigh the risks of CI-AKI. This also applies to patients with abnormal chest X-ray, of whom a high proportion will have non-diagnostic V/Q scans. While bilateral ultrasound dopplers can also be considered, with no further imaging in the event of a DVT, the yield of lower-extremity ultrasound in patients without suggestive clinical features is likely low. Patients at risk for CI-AKI who undergo CT angiography should receive appropriate preventative measures before contrast exposure in consultation with experts in nephrology as required. Low dose contrast protocols have been shown to be non-inferior in small studies and should also be considered in at-risk patients [70]. It should also be noted that in patients who are dialysis dependent and unlikely to improve, the risk of further renal deterioration is minimal and CTPA can be utilized in consultation with Nephrology.

A SPECT V/Q scan is performed the next morning and is interpreted as positive for right-sided pulmonary embolism.

Despite the widespread use and increased radiographic resolution, the clinical impact of SPECT over planar V/Q scan is an area of ongoing research [7]. Specifically, it remains unclear whether patients with positive SPECT V/Q scans and negative planar V/Q scan have improved outcomes with anticoagulation. This same question is also being asked for patients diagnosed with sub segmental pulmonary embolism on CT imaging [71]. Up to 1/6 patients with negative V/Q scans and 1/4 with negative D-dimer tests may have subsegmental PEs [72,73]. In addition, there is considerably lower interobserver (radiologist) agreement for subsegmental PEs [74].

## 3. Conclusions

### 3.1. Areas of Future Research

The current paradigm for VTE diagnosis provides a variety of tools but also leaves many unanswered questions. In particular, what clinicians need most is better information about *recurrence*—that is, diagnostic methods that more accurately predict which patients are at risk for further thrombotic events and require aggressive anticoagulation. With this in mind, we outline three pressing questions and describe ongoing research within each domain.

#### 3.1.1. How to Reduce Overuse of Imaging Tests and Overdiagnosis of VTE?

The pinnacle of VTE diagnosis is a bloodless and imaging-free approach that can reliably rule out thrombosis and identify patients at low risk of recurrent events. The PERC rule, which can identify low-risk patients in whom PE can be ruled out on clinical examination/history without further testing, exemplifies this approach but is only applicable to a small subset of patients [7]. Future research that can apply similar prediction rules to a greater proportion of patients is much needed, especially for medical inpatients where there is a significant burden of laboratory and radiographic utilization. The increased use of machine learning and artificial intelligence is one avenue for innovation. Such approaches will require large sets of well-ordered data, highlighting the need for global cooperation and standardization in data collection. However, even in a blue sky scenario, many patients will require further diagnostic evaluation with laboratory tests and imaging studies. The poor specificity of D-dimer tests and potential for harm and overdiagnosis with CT scans is therefore an area that must also be addressed. Studies that are specifically examining D-dimer include ADJUST-DVT (NCT02384135) and 4D (NCT02038530). Novel biomarkers for VTE such as P Selectin are also being evaluated [17].

#### 3.1.2. How to Risk Stratify Patients with Venous Thrombosis at Distal Sites?

Improved resolution and access to diagnostic imaging has led to a higher than ever incidence of VTE. Yet not all venous thrombotic events are created equally. For example, as illustrated by our cases, what to do with isolated distal DVT and subsegmental PEs remains a muddy situation with arguments both for and against anticoagulation. A better understanding of the diagnostic factors that predict which patients with distal VTE remain at risk for recurrence is a research priority. Clinical prediction rules and algorithms that specifically address these groups are highly anticipated. Two approaches that are currently undergoing study include withholding anticoagulation in patients with subsegmental PE and negative lower-extremity ultrasounds (NCT01455818) and completing screening CTPA in patients with IDDVT and elevated D-dimer values.

#### 3.1.3. How to Optimally Diagnose VTE in Patients with Elevated Baseline Pretest Probability?

The majority of existing diagnostic tools are intended for the general population and do not always apply well to subgroups at high risk for thrombosis. Within this paper, we explored how diagnostic thresholds differ during pregnancy and for those with a history of VTE. This is also pertinent to the cancer population. The gap in VTE cancer diagnosis was highlighted in a recent paper which showed that clinical prediction rules and D-dimer strategies were unable to rule out VTE in the vast majority of cancer patients [75]. The clinical utility of both specialized clinical prediction rules/D-dimer strategies as well as “imaging-first” approaches continue to be evaluated.

A further unique aspect for patients with previous VTE is how to interpret imaging. For most cases, determining the age of a radiographically confirmed VTE is more of an art than a science and there is little recourse for the patient without prior scans for comparison. Research that examines the changing composition of venous thrombosis over time may be able to shed new light. While not visible with traditional imaging modalities, MRI imaging can identify changes in thrombus methemoglobin concentrations over time [76]. This approach is now being prospectively validated in the THEIA study (NCT02262052).

## Figures and Tables

**Table 1:** Overview of clinical and laboratory prediction rules for suspected DVT and PE.

Prediction Rule.	Sensitivity (95% CI)	Specificity (95% CI)	Notes
Wells PE Score (Cut Off ≤ 4) [10]	0.60 (0.49–0.69)	0.80 (0.75–0.84)	Developed and best validated for use in outpatients (subspecialty clinics and emergency departments) with suspected pulmonary embolism. Alternative version uses three levels of stratification.
Wells DVT Score [11]	0.56 (0.51–0.63)	0.88 (0.85–0.91)	Developed and best validated for use in patients with suspected first DVT presenting to outpatient subspecialty setting.
Revised Geneva Score [10]	0.91 (0.73–0.98)	0.37 (0.22–0.55)	Developed in patients with suspected PE admitted to the emergency department. Validation populations included greater proportion of outpatients.
LEFt Score [12]	1.00 (0.81–1.00)	0.50 (0.43–0.58)	Developed in pregnant woman with suspected first-time DVT.
YEARS Score [13]	0.97 (0.91–99.7)	0.14 (0.12–0.17)	Developed in patients with suspected PE who were largely outpatients but inpatients not excluded. Validated in the emergency department.
Standard D-Dimer Interpretation [14]	0.99 (0.98–1.00)	0.45 (0.40–0.51)	D-dimer applied to patients with low or low/intermediate clinical pretest probability.
PTP-Adjusted D-Dimer Interpretation [14]	0.99 (0.97–1.00)	0.57 (0.49–0.65)	
Age-Adjusted D-Dimer Interpretation [14]	0.98 (0.96–1.00)	0.55 (0.48–0.61)	
